# Mesenchymal Stromal Cells as a Cellular Target in Myeloid Malignancy: Chances and Challenges in the Genome Editing of Stromal Alterations

**DOI:** 10.3389/fgeed.2020.618308

**Published:** 2021-01-22

**Authors:** Bella Banjanin, Rebekka K. Schneider

**Affiliations:** ^1^Department of Hematology, Erasmus Medical Center Cancer Institute, Rotterdam, Netherlands; ^2^Oncode Institute, Erasmus Medical Center Cancer Institute, Rotterdam, Netherlands; ^3^Department of Cell Biology, Faculty of Medicine, Institute for Biomedical Engineering, Rheinisch-Westfälische Technische Hochschule (RWTH) Aachen University, Aachen, Germany

**Keywords:** genome-editing, BM MSCS, myeloid maliganancies, stromal alterations, BM niche

## Abstract

The contribution of bone marrow stromal cells to the pathogenesis and therapy response of myeloid malignancies has gained significant attention over the last decade. Evidence suggests that the bone marrow stroma should not be neglected in the design of novel, targeted-therapies. In terms of gene-editing, the focus of gene therapies has mainly been on correcting mutations in hematopoietic cells. Here, we outline why alterations in the stroma should also be taken into consideration in the design of novel therapeutic strategies but also outline the challenges in specifically targeting mesenchymal stromal cells in myeloid malignancies caused by somatic and germline mutations.

## Introduction

**Figure 1:**
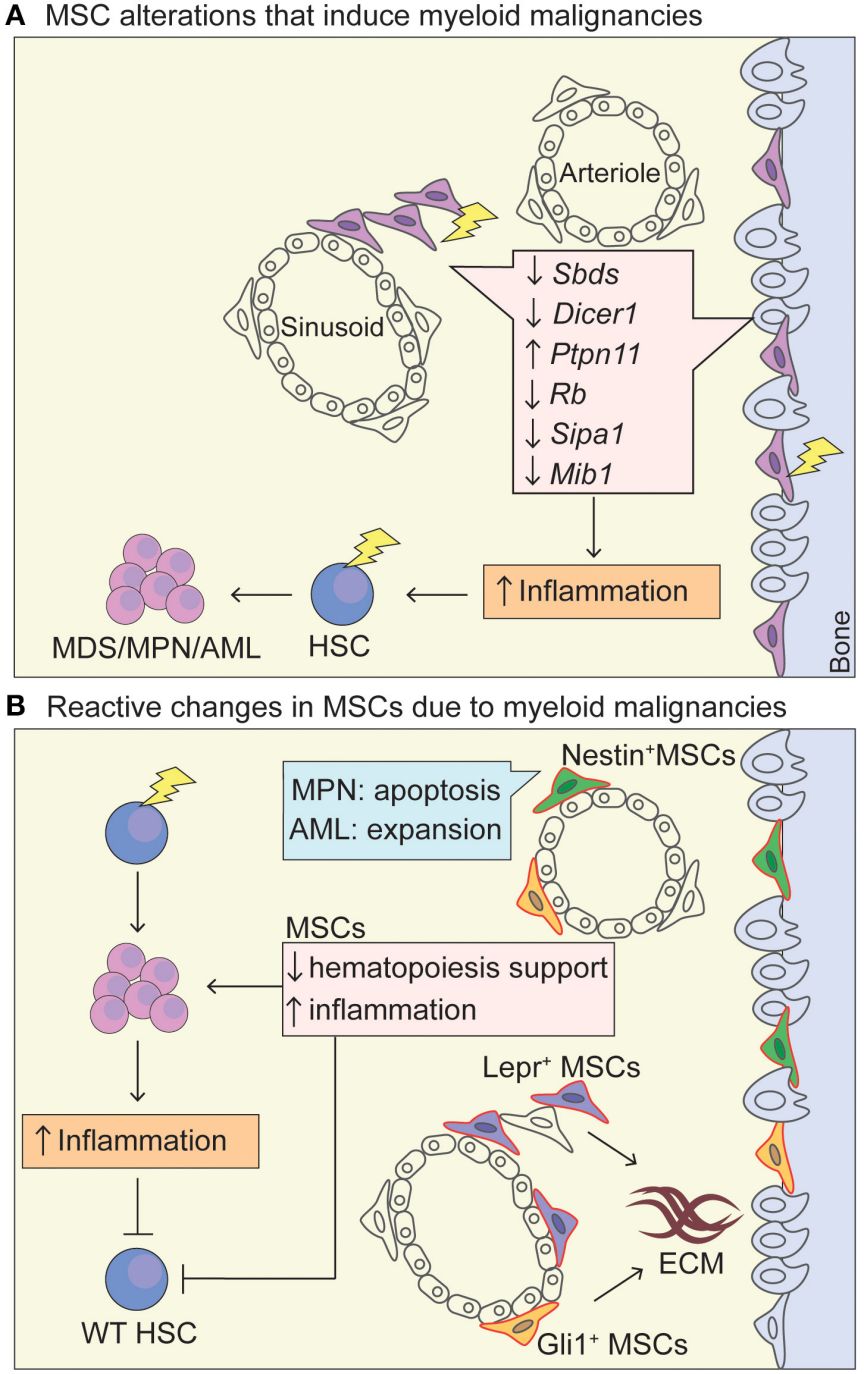
MSC niche in myeloid malignancies. **(A)** Schematic representation of MSC niche alterations that promote the leukemic transformation. Lightning bolt indicates genetic lesion in MSC-like cells that have been shown to promote oncogenesis through increased inflammation and increased (genotoxic) stress of HSCs. Deletion of *Dicer1* and *Sbds* in Osterix^+^ osteoprogenitor MSCs leads to a MDS phenotype with sporadic AML upon *Dicer1* deletion (Raaijmakers et al., 2010; Zambetti et al., 2016). Activating mutations in tyrosine-protein phosphatase non-receptor type 11 (*Ptpn11*) in Nestin-Cre^+^ cells induces a MPN phenotype. A MDS/MPN phenotype was also seen in activating *Ptpn11* in *Mx1-*Cre^+^, *Prx1-*Cre^+^, *Lepr*-Cre^+^, *Osx1*-Cre^+^ cell-type specific knock-in mice, highlighting that MSCs and osteoprogenitors can induce MPN and that there is probably overlap in cells populations targeted by the Cre-drivers, whereas differentiated osteoblasts (Oc-Cre^+^) and endothelial cells (VE-Cadherin-Cre^+^-ERT2) could not induce MPN (Dong et al., 2016). *Rb* (encodes RB protein) (Walkley et al., 2007) and *Mib1* (encodes mind bomb 1 protein) (Kim et al., 2008) promote an MPN-like phenotype in a Mx1-Cre^+^ driver. The Mx1-Cre^+^ driver traces MSC-like cells that are located within the bone marrow but also at the periosteum (Ortinau et al., 2019), and have limited *in vivo* adipogenic differentiation potential, making them more osteoprogenitor-like (Park et al., 2012). *Sipa1* expression is most abundant in CD31^+^ BM endothelial cells, but also found in MSCs (CD45^−^Lin^−^CD31^−^CD51^+^Sca1^+^) (Xiao et al., 2018). Deletion of *Sipa1* results in the development of MDS/MPN and *Sipa1*^−/−^ MSCs cultured *in vitro* show increased adipogenic and chrondrogenic differentiation potential, but impaired osteogenic differentiation (Xiao et al., 2018). **(B)** Upon exposure of a mutated hematopoietic cell within the niche, MSCs are functionally altered through cytokine stimulation, direct cell-cell contact and activation of inflammatory pathways, promoting the survival of the mutant HSC in favor of WT HSCs and increase in inflammatory signatures. Specifically, Lepr-Cre^+^ (Decker et al., 2017) and Gli1-Cre^+^ (Schneider et al., 2017) cells expand and proliferate in an MPN setting and produce extracellular matrix. Nestin-Cre^+^ cells proliferate in AML and provide chemotherapy resistance (Forte et al., 2020), whereas they become apoptotic in MPN disease due to neural damage and Schwann cell death triggered by interleukin-1β production by the mutated HSC. AML cells seem to induce osteogenic differentiation and block adipogenesis of MSCs, as well as blocking maturation of osteolineage-MSCs into mature osteoblasts (Battula et al., 2017; Pievani et al., 2020b). MSCs, mesenchymal stem cells; HSC, hematopoietic stem cells; AML, acute myeloid leukemia; MPN, myeloproliferative neoplasm; MDS, myelodysplastic syndrome; ECM, extracellular matrix; Lepr, Leptin receptor; Prx1, Paired related homeobox 1; Mx-1, Myxovirus Resistance-1.

Under physiological conditions, hematopoietic stem cells (HSCs) are regulated by their bone marrow microenvironment (BMM) through cellular interactions and secreted factors to maintain a continuous pool of hematopoietic cells (Morrison and Scadden, 2014; Pinho and Frenette, 2019). This crosstalk between the hematopoietic system with its surroundings is essential for the proper functioning of HSCs throughout life and becomes deregulated in hematological malignancies. The main constituents of the BMM are bone marrow mesenchymal stromal cells (MSCs), osteolineage cells (OLCs), endothelial cells, amongst various other cells including adipocytes, neural, and hematopoietic cells (Pinho and Frenette, 2019; Méndez-Ferrer et al., 2020). MSCs are a heterogenous group of non-hematopoietic cells that express key hematopoiesis-supporting factors such as stem cell factor (SCF) and CXC motif ligand (CXCL)-12. In humans the surface markers CD271 and CD146 have been shown to enrich for cells that can form fibroblast colonies (CFU-F) (Kfoury and Scadden, 2015). MSCs have been described in mouse models using numerous Cre-drivers and surface markers outlined in Figure 1.

Historically, the development of myeloid malignancies was considered to be HSC-intrinsic, be it driven by germline or somatic mutation. The BMM can either facilitate oncogenesis by supporting the expansion of malignant cells, and suppressing normal hematopoiesis, or induce oncogenesis by acquiring mutations or functional alterations that pre-dispose for oncogenesis. These two theories are not mutually exclusive, as is amply exemplified in the pathogenesis of myeloid malignancies including myeloproliferative neoplasms (MPN), myelodysplastic syndromes (MDS) and acute myeloid leukemia (AML) (Medyouf, 2017; Fathi et al., 2019; Behrmann et al., 2020). Thus, the mutual interaction between mutated HSCs and the BMM has further evolved as an attractive novel therapeutic target.

In this article, we will outline the role of stromal cells (specifically BM MSCs) in myeloid malignancies in somatic disease, as well as germline conditions, and describe recent progress in dissecting the HSC-stroma crosstalk. Finally, we discuss possible application of the established murine disease models and future challenges in developing genetically targeted therapies for the BM stroma.

## The Role of the Stroma in Leukemia Pre-Disposition Syndromes

The World Health Organization (WHO) introduced a new category of “myeloid malignancies with germline predisposition” to the 2016 Classification of hematopoietic tumors. Given that these “rare” mutations are only coming to light with increased use of parallel sequencing platforms in population and family studies (Porter, 2016; Miller et al., 2018; Kim et al., 2020), it can be speculated that germline stromal mutations exist which have yet to be discovered. An exemplary disease is Shwachman-Diamond syndrome (SDS); a rare autosomal recessive bone marrow failure disorder caused by mutation in the *SBDS* gene with a cumulative probability of leukemic progression of >30% at the age of 30 years (Dale et al., 2006; Nelson and Myers, 2018). Hematopoietic cell specific deletion of *Sbds* did not result in MDS or AML in two murine disease models (Rawls et al., 2007; Zambetti et al., 2015), whereas exposure of wildtype HSCs to *Sbds*-deficient osteolineage MSCs led to an MDS phenotype and genotoxic stress in HSCs (Zambetti et al., 2016). The prominent clinical feature of skeletal abnormalities in SDS patients was recapitulated through the niche-specific deletion of *Sbds* (Zambetti et al., 2016). Importantly, the alarmin heterocomplex S100A8/9 secreted by the niche was identified as a candidate driver of inflammatory stress in HSCs, highlighting that the crosstalk between stroma and HSCs is of particular interest as a possible therapy target. Targeted deletion of *Dicer1* in osteolineage MSCs resulted in reduced expression of *Sbds* in transplanted WT HSCs (Raaijmakers et al., 2010). The resulting phenotype displayed key features of human MDS and a tendency to develop AML; clearly showing that alterations in BM stromal cells can induce malignancy and stress in HSCs (Raaijmakers et al., 2010). Intriguing case studies of donor cell-derived leukemia (DCL) development upon allogeneic HSC transplantation in humans have brought about the possibility of oncogenesis driven by the diseased recipient BMM (Berger et al., 2016; Engel et al., 2018).

In line, numerous genetic modifications (deletions) in non-hematopoietic, stromal cells were reported to give rise to a myeloproliferative phenotype *in vitro* and *in vivo* (Rupec et al., 2005; Walkley et al., 2007; Xiao et al., 2018), but also activation of e.g., Notch signaling (Kim et al., 2008; Dong et al., 2016). Inflammation seems to play an important role in the pathogenesis of these myeloid malignancies. As an example, IL-1B propagates an inflammatory BMM as it activates HSCs to differentiate toward myeloid cells and monocytes (Rupec et al., 2005). Early stages of MPN disease are also characterized by increased IL-1β expression, which triggers pro-inflammatory damage to the BMM and advances disease progression (Arranz et al., 2014). This showcases that stromal drivers influence the hematopoietic system and can result in secondary neoplasms (schematically depicted in Figure 1A).

## Crosstalk Between Non-Mutated Stroma and Hematopoietic Cells With Somatic Mutations

It is becoming evident that the BMM is functionally altered by exposure to hematopoietic cells harboring somatic mutations, creating a proinflammatory environment that seems to propagate leukemic disease development and supresses normal hematopoiesis (Figure 1B). Overall differences in MSC compartments have been noted in myeloid malignancies compared to normal bone marrow. In AML, there is generally a reduction of bulk MSCs. However, Nestin^+^ cells, as well-documented MSCs in the BM (Mendez-Ferrer et al., 2010), have been shown to be 4–5-fold more abundant in human AML patients, in line with expansion of Nestin^+^ cells in the murine iMLL-AF9 AML model (Hanoun et al., 2014; Forte et al., 2020). This is in striking contrast to the decrease in Nestin^+^ cells in murine models and human MPN (Arranz et al., 2014; Drexler et al., 2019), suggesting that the same group of niche cells can behave differently in various myeloid malignancies and/or stages of leukemic disease. Conditional depletion of Nestin^+^ cells upon AML development in iMLL-AF9 mice lead to a significantly extended mouse survival, suggesting that Nestin^+^ cells promote leukemogenesis *in vivo* (Forte et al., 2020). Importantly, in a competitive transplant setting, depletion of Nestin^+^ cells during AML development selectively diminished the number of leukemic cells, while leaving normal hematopoiesis unaffected (Forte et al., 2020), which is one of the major challenges in the treatment of AML.

The direct effect of a mutated hematopoietic clone on the bone microenvironment is evidently illustrated in murine models but also patient samples with bone marrow fibrosis. In a murine model of CML, endosteal OLCs expanded upon expression of BCR/ABL in the hematopoietic compartment leading to deposition of extracellular matrix (Schepers et al., 2013). In response to MPN clones, Gli1^+^ stromal cells are activated from their normal endosteal and perivascular niches and significantly expand in murine models and patient samples (Schneider et al., 2017). Importantly, their genetic ablation ameliorates fibrosis, proving functional proof that they play a central role in the fibrotic transformation. Another stromal subset of Lepr^+^ MSCs has been shown to expand in fibrosis (Decker et al., 2017). Conditional deletion of platelet-derived growth factor receptor a (Pdgfra) from Lepr^+^ cells or the administration of the tyrosine kinase inhibitor imatinib suppressed Lepr^+^ cell expansion and mitigated fibrosis. There seems to be a common initial pro-inflammatory stromal response to the malignant MPN clone that poises the stroma to become pro-fibrotic (Gleitz et al., 2020; Leimkuhler et al., 2020). This is in line with the observation of a diseased niche characterized by cellular stress and an increased inflammatory signature in bulk RNA-sequencing of prospectively sorted mesenchymal cells from human low-risk MDS patients (Chen et al., 2016). Additionally, human MDS MSCs produce inflammatory cytokines (IL-1β, IL-6, and TNFα) compared to control *in vitro* cultured MSCs (Flores-Figueroa et al., 2002, 2008). Notably, IL-6 knockout in the BM reduces fibrosis in a MPN setting (Gleitz et al., 2020). Likewise, our group demonstrated increased expression of the inflammatory S100A8 alarmin in the stromal niche in murine models and patient samples of del(5q) MDS (Ribezzo et al., 2018). This increased expression of S100A8 in MSCs resulted in decreased hematopoiesis-support *in vitro*, indicating that mutated hematopoietic cells can initiate a vicious cycle of inflammation in the niche, leading to decreased support of normal hematopoiesis and fuelling the progression of haematopoietic malignancy. The common denominator in hematological malignancies driven by somatic or germline mutations thus seems to be an inflammatory “mutagenic” microenvironment that precedes malignant transformation and disease progression (Craver et al., 2018; Gleitz et al., 2018; Leimkühler and Schneider, 2019; Pronk and Raaijmakers, 2019).

## Challenges Of Genetic Editing in the Bone Marrow Stroma

As outlined, the bone marrow stroma seems to play a significant role in the initiation, maintenance and progression of myeloid malignancies and murine models indicate that MSCs are a highly attractive therapeutic target. The importance of targeting the stroma is highlighted by the fact that despite improvements in the treatment of AML, long-term survival is <30% in adults (Ferrara and Schiff, 2013). In murine models, specific subsets of stromal cells can be modified by using stromal Cre-drivers. The correlate to this procedure in the human setting would optimally be genome editing of stromal cells. Nuclease-based site-specific genome editing has provided an unprecedented opportunity to artificially modify genetic information within mammalian cells (Romito et al., 2019). The clustered regularly interspersed short palindromic repeats (CRISPR)/Cas9 system has been used to create germline and somatic mouse models, and has the benefits of relatively easy design and high mutational efficiency (Mou et al., 2015; González-Romero et al., 2019; Broeders et al., 2020; Lee et al., 2020). The HSC has been the most relevant cell type to edit, with major advances in Cas9 clinical translation made, particularly in the monogenetic disorders sickle cell disease and β-thalassemia (Dever and Porteus, 2017).

In this section we highlight some of the key challenges hampering the development of targeted genetic therapy of BM stromal populations: (1) identification of specific MSC population to edit, (2) targeting MSCs in their *in situ* location vs. *ex vivo*, (3) indirect targeting of MSC function *in vivo* through genome editing of hematopoietic cells and cell-to-cell interactions, and (4) *in vitro* functional characterization of MSCs and potential therapeutic targets through CRISPR screens and 3D models.

## Identification and Targeting MSCs *In Situ* – Direct Vs. Indirect Strategies

Much of our understanding of the BM MSCs has originated from genetic-fate tracing mouse models in which MSC populations have been labeled via a stromal Cre-driver (Kfoury and Scadden, 2015). Functionality of these Cre-drivers has been shown by conditional deletion using diphteria-toxin receptor based mechanisms (Schneider et al., 2017; Pinho and Frenette, 2019). Additionally, Cre-drivers of MSC populations provide spatial information when combined with a fluorescent-reporter. Nevertheless, the current widely-used Cre-drivers likely label heterogeneous groups of MSCs, outlined in a recent review (Al-Sabah et al., 2020). The recombination efficacy in Cre-drivers has resulted in variable results (Chen et al., 2017), while conditional Cre-lines result in higher specificity compared to constitutive Cre-lines and allow fate-tracing experiments in health and disease setting (Méndez-Ferrer et al., 2020). Recent advancements in single-cell RNA sequencing (scRNAseq) have for the first time allowed us to zoom in on heterogeneous populations within the murine BMM (Schroeder et al., 2016; Baryawno et al., 2019; Tikhonova et al., 2019; Wolock et al., 2019; Baccin et al., 2020; Leimkuhler et al., 2020). Tikhonova et al. have shown that the Lepr^+^ Cre-driver previously studied as one MSC population, contains four subclusters of MSCs, with functional differences between them as current evidence suggests. As we gain knowledge of functionally distinct MSCs and their possibly common progenitors, it will become possible to target them. Importantly, the location of MSCs in relation to (mutant) HSPCs seems to predict biological functionality and these sinusoidal and CXCL12 niches will need to be further investigated (Gomariz et al., 2018; Baccin et al., 2020; Kokkaliaris et al., 2020). Perhaps the use of multiplexed imaging (Kokkaliaris et al., 2020) in combination with laser-capture techniques to isolate specific BM populations (Baccin et al., 2020) can aid in inferring spatial and signaling relationships between cells from single cell transcriptomic data.

Specifically, these new techniques can help identify new druggable pathways through, for example, ligand-receptor analysis between mutated hematopoietic cells and the stromal counterpart in myeloid malignancies (Efremova et al., 2020). This method was very recently employed in the unbiased scRNAseq paper showing populations of murine and human MSCs interacting with hematopoietic populations in MPN (Leimkuhler et al., 2020). A druggable alarmin axis was identified in the fibrotic transformation both in murine models and patients and treatment with Tasquinimod, inhibiting the binding of the alarmins S100A8/S100A9 to TLR4, ameliorated the MPN phenotype in mice.

Due to the lack of evident genetic modifications and a prominent cell of origin, a clear-cut molecular target for BM MSCs is not apparent. It is possible, however, to target HSPCs as they are relatively easily accessible for genome editing. The use of gene therapy for neurometabolic disorders using HSPC transplantation has shown that overexpression of therapeutic proteins has cross-correction capacity as also non-hematopoietic cells are being exposed to the therapeutic effect (Ferrari et al., 2020). This could be useful if loss-of-function mutations are found in MSCs.

One could imagine that mutated hematopoietic cells can be examined for specific receptors that are not vital for their physiological function but are unique for their malignant interaction with stroma (Kokkaliaris and Scadden, 2020; Pievani et al., 2020a). The α4β1 integrin–VCAM1 axis between stroma and the AML mutant cell aids in chemoresistance (Jacamo et al., 2014; Carter et al., 2016). AML chemo-resistant cells also have high expression of very late antigen 4 (VLA4) which facilitates adherence to the stroma through VCAM1 activated NF-kB signaling (Jacamo et al., 2014). Indeed, patients with VLA-4-negative AML have a more favorable prognosis, highlighting the role of stroma-HSCPs cross-talk (Matsunaga et al., 2003). Within the CXCL12-CXCR4 axis, CXCL12 is expressed by MSCs and interacts with HSCs via the binding to CXCR4, regulating their mobilization (Greenbaum et al., 2013). Blockade of this axis can release leukemic cells from their chemoprotective niches (Nervi et al., 2009). Recently, an elegant *in vivo* pooled CRISPR screen targeting selected cell surface genes was performed in murine *MLL-AF9* AML cells and identified CXCR4 as a positive regulator of leukemic cells, indispensable for their growth and survival *in vivo* (Ramakrishnan et al., 2020). CXCR4 is essential for the development of AML independently of its interaction with CXCL12 on MSCs or endothelial cells. In contrast, *Cxcr4*^−/−^ normal HSCs are capable of long-term hematopoiesis (Nie et al., 2008), highlighting the different biology in homeostasis and malignant disease and possible targeting avenues.

As an example, inflammation within the BM niche, specifically the erythroblastic niche, can be targeted by genetically editing the hematopoietic cell. In our previous work, we applied CRISPR-Cas9 technology in a murine MDS model to genetically inactivate *S100a8* and improve the defective erythropoiesis characteristic for the disease. Compared to control non-targeting sgRNA, CRISPR-mediated inactivation of *S100a8* in MDS cells restored erythropoiesis and restored a normal erythroid niche by interrupting the cycle of inflammation (Schneider et al., 2016).

Ideally, the complex interplay between the hematopoietic system and the stroma could be modeled more efficiently with CRISPR-Cas9 based techniques in mice (Heckl et al., 2014; Tothova et al., 2017). With advancements in deep-sequencing, novel germline/somatic mutations in stroma of patients might be identified. More complex models could then be made to mimic the different mutations identified in the hematopoietic and the stromal compartment in mice, to search for druggable targets. The genome-editing efficiency is also consistently being improved, with DNA-free systems being developed that are more suitable for human trials as there is no risk for random insertional mutagenesis (Shapiro et al., 2020).

## Genetic Editing of Stromal Cells *Ex Vivo*: Feasibility of Delivery

A commonly used CRISPR/Cas9- based technique for gene editing *ex vivo* is the isolation of the target cell and delivery of the gene-editing machinery via electroporation, microinjection, or virus-based vehicles before injecting the corrected cells back into patients or mice (Broeders et al., 2020). MSCs in general have been widely investigated for use in multiple diseases due to the ease of their isolation (plastic adherence and self-renewal properties), their low immunity potential, and their ability to secrete factors (Kean et al., 2013). The production of inflammatory cytokines such as PDGF, TNFa, CCR8, and CCR2 within the solid organ tumor microenvironment, has been shown to enhance homing of MSCs to the tumor location (Marofi et al., 2017). Primary MSCs can express CRISPR/Cas9 proteins through nucleofection, lentivirus, and non-integrating adeno-associated virus (Golchin et al., 2020). However, the homing of edited MSCs to the bone marrow niche has not been formally tested yet.

A possible technique by which CRISPR-based strategies on the BM stroma could be performed, is by injecting complete CRISPR-proteins through intrafemoral injections. Intrafemoral injection has been used to model osteosarcoma in orthotopic mouse models (Sasaki et al., 2016). The only downside is that off-target effects on surrounding (hematopoietic) cells can occur. To circumvent this, a possibility could be to expand MSCs *ex vivo* and genetically alter them using CRISPR *in vitro*, and then inject them back through an intrafemoral injection. It has been shown that donor MSCs injected via intramarrow injection also contribute to the reconstitution of the stromal niche in the ablated bone marrow of recipient mice (Muguruma et al., 2006; Ahn et al., 2010; Zhou et al., 2014). Intraosseal therapy could pose clinical challenges, with an invasive procedure that has an increased chance of complications (in particular infections), compared to intravenous or intraarterial administration. However, the intravenously administered MSCs easily get trapped in the lung circulation and have limited engraftment of about a week, whereas arterially administered MSCs seem to engraftment better at the site of injury, e.g., hind leg bone irradiation in mice (Kean et al., 2013). First, proof of principle studies using intrafemoral/intraosseal injections need to be performed where candidate genes can be knocked out or mutations introduced within the mouse or even specifically in the stroma by using floxed Cas9 mice crossed to specific stromal Cre-drivers. The beauty of this method in mice is additionally that one leg can be edited while one leg serves as a non-targeted control. A major point to consider, however, is the determination of recombination efficiency within the bone marrow stroma as MSCs are difficult to obtain as single-cell suspension cells. A possible read-out here could be *in situ* hybridization of mRNA of the targeted genes in a multiplex imaging set-up.

Cas proteins need specially designed delivery vehicles for tissue-specific delivery as they cannot cross biological barriers themselves and have a high positive charge and molecular mass. Extracellular vesicles (EVs) are used as possible packing devices for sgRNA:Cas9 ribonucleoprotein complexes. It has been shown however that EVs are mainly taken up by the liver (~84%), whereas roughly 1.6% are found back in the bone marrow 4 h upon systemic administration, making delivery to the bone marrow quite challenging (Kostyushev et al., 2020). Progress is made on engineering functionalized exosomes (M-CRISPR-Cas9 exosome) which encapsulate CRISPR-Cas9 components more efficiently (Ye et al., 2020). Recently, the interest for vesicle nanoparticles containing the Cas9 machinery has been growing. While traditionally nanoparticles can mainly be found in the liver and lung after injection, a recent breakthrough study (Krohn-Grimberghe et al., 2020), reported the design and *in vivo* performance of systemically injected lipid–polymer nanoparticles encapsulating small interfering RNA (siRNA), for the silencing of genes specifically in bone-marrow endothelial cells. Using nanoparticle enabled RNAi, the group targeted stromal-derived factor 1 (*Sdf1*) resulting in stem cell liberation into the blood, and monocyte chemotactic protein 1 (*Mcp1*) whose silencing retained monocytes in the BM. These modified nanoparticles lay the ground for editing non-hematopoietic cells in the bone marrow with a high efficacy and show that HSPCs biology can be altered through stroma alterations.

## Functional Testing of Genetic Modification of MSCs *In Vitro* and *In Vivo*

Isolation of MSCs directly from the BM remains a challenge as stromal cells are closely associated with extracellular matrix within the marrow and single-cell suspensions are difficult to obtain even after digestion of bone (Gomariz et al., 2018). Most often, MSCs are left to grow out from bone chips or human aspirates and selected for on the basis of their plastic adherence. Cultured human MSCs are minimally characterized by their trilineage differentiation potential, expression of surface markers that enhance CFU-F potential, and plastic adherence *in vitro* (Dominici et al., 2006; Kfoury and Scadden, 2015; Agha et al., 2017). Murine MSCs are often identified by a panel of typical surface markers and have a less stringent definition (Agha et al., 2017). Nevertheless, functional characterization of *in vitro* isolated cells still needs to be optimized, as even a short-term (passage 0) *ex vivo* culturing environment greatly reprograms MSCs compared to direct sorting of primary cells for microarray analysis (Ghazanfari et al., 2017). Despite retaining their *in vitro* clonogenicity and tri-lineage differentiation potential (Pevsner-Fischer et al., 2011), culture-induced gene expression changes are present and raise the question of comparability of primary and cultured cells, as well as the possibility that only specific subsets of MSCs are selected for in adherent culture (Tormin et al., 2009). BM MSCs cultured as non-adherent 3D sphere colonies termed mesenspheres, have been reported to retain MSC surface markers, tri-lineage potential, and to have an increased self-renewal potential in serial transplantations into immunodeficient NOD scid gamma mice compared to adherent cultured cells (Ghazanfari et al., 2016). Gene expression in cultured MSC mesenspheres was still altered compared to primary sorted MSCs, but 3D cultured cells had more osteogenic and adipogenic transcription factor expression compared to 2D adherent cells (Ghazanfari et al., 2017). This difference in culturing conditions might be confounding as Forte et al. have shown that in MSCs derived from the same AML donors, only mesenspheres provided enhanced chemoprotection of human AML blasts, whereas plastic-adherent MSCs did not (Forte et al., 2020). The improved fitness of 3D cultured MSCs advocates for its use. Ideally, there will be a standardized protocol for the isolation of murine and human BM MSCs so that results from different groups can be compared easily (Stroncek et al., 2020).

Patient derived cultured MSCs however, in 2D but also 3D cultures, can serve as a platform for personalized screening approaches to detect alterations which hamper therapy or find potential targets. As an example, a genome-scale CRISPR knock-out screen was used to uncover imatinib-sensitizing genes *in vitro* on K562 cells (Lewis et al., 2020). Although this was performed on cell lines, one can imagine broadening the application and do similar tests in smaller format (due to the high cell number needed) on patient derived cells.

These methods could be used as proof-of-principle platforms to identify candidate proteins for genome editing. Recently, human mesenchymal stromal cells were shown to endure nucleofection with Cas9-adeno-associated virus serotype 6 (AAV-6) and genome-editing including gene disruption and targeted integration of up to 3.2 kb of DNA with stable transgene expression, while retaining their *in vitro* tri-lineage differentiation potential and phenotypical signature (Srifa et al., 2020). Through integration of PDGF-BB, VEGFA, and IL-10 transgenes at the *HBB* locus they successfully created hypersecreting hMSC which actively improved wound healing in diabetic wounds of mice. Specifically the combination of scaffolds coated with human MSCs could be modified with the Cas9-AAV-6 system to model normal and malignant human hematopoiesis by subcutaneous implantation in immunodeficient mice (Vaiselbuh et al., 2010; Abarrategi et al., 2017; Passaro et al., 2017). The benefit of such a system is that patient-derived leukemic cells can grow in the hMSC scaffolds as they form ectopic humanized BMM and can be followed up for long periods of time in an *in vivo* setting. Similarly, human femur-derived bone fragments from AML patients were transplanted into NSG mice using Matrigel as a carrier and were vascularized 4 weeks post implantation (Battula et al., 2017). These systems could allow for easily-accessible and controllable *in vivo* gene-editing of multiple relevant human BMM populations in the presence of clonal xenografted AML cells.

## Future Outlook

As we gain knowledge of the different functional subcomponents of the bone marrow niche, the disease model of myeloid malignancies will become more complex. It is evident that oncogenesis can arise from two non-mutually exclusive theories: niche-induced and niche-facilitated. In patients, we envision a future of personalized medicine in which the stroma can be pharmacologically targeted in combination with a hematopoietic cell-based therapy. We can use the accumulating knowledge with genome editing by (1) generation of murine disease models *in vivo* on the basis of new possible germline/somatic mutations within the niche found with targeted sequencing in human disease to study disease pathogenesis, (2) targeting MSCs *in vivo* directly through MSC/EV-based approaches, (3) indirectly through modulation of hematopoietic cells, (4) modeling of the human hematopoietic niche using ossified scaffolds in xenotransplantations, and (5) *in vitro* Cas9-based screening methods. Targeted genome-editing will most likely become more feasible as we characterize the true MSCs as the target cell and improve engineering of carriers which will deliver the sgRNA:Cas9 cargo with high efficacy to the bone marrow.

## Author Contributions

All authors listed have made a substantial, direct and intellectual contribution to the work, and approved it for publication.

## Conflict of Interest

The authors declare that the research was conducted in the absence of any commercial or financial relationships that could be construed as a potential conflict of interest.
